# Supplementary material for: Four-Color Pseudovirus-Based Neutralization Assay: A Rapid Method for Evaluating Neutralizing Antibodies Against Quadrivalent Hand, Foot, and Mouth Disease Vaccine
Source: Vaccines (Basel). 2025 Mar 18;13(3):320. doi: 10.3390/vaccines13030320 (PMC11946612; doi:10.3390/vaccines13030320)
Supplement: Supplementary file 1 [file vaccines-13-00320-s001.zip › vaccines-3501239-supplementary.pdf]

Supplementary Materials

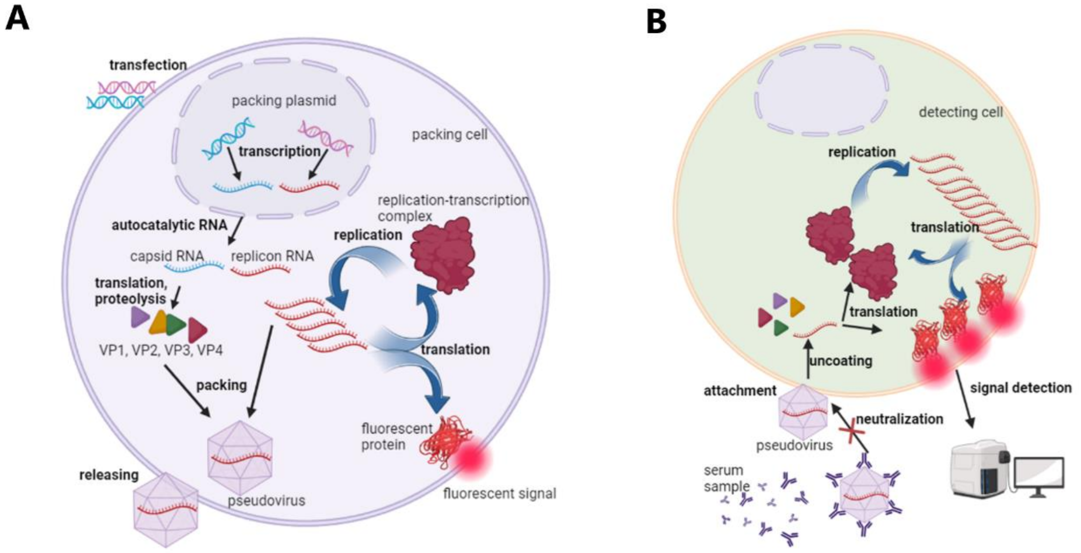

Figures S1. (**A**) Principle of enterovirus pseudovirus packaging and neutralization mechanism. The engineered viral genome is divided into two components: the replicon and capsid expression gene. The replicon expression gene is transcribed into replicon RNA within the host cell, which is subsequently translated to produce the corresponding replicase and the incorporated fluorescence reporter gene. The capsid protein, expressed by the capsid expression gene, self-assembles into virus-like particles that encapsulate the replicon RNA, ultimately forming pseudoviruses capable of single-cycle infection and carrying fluorescent protein reporter genes. (**B**) Neutralization mechanism. Fluorescent pseudoviruses mimic the process of cell infection by authentic viruses. Prior to pseudovirus entry into the cells, the addition of neutralizing serum can inhibit the pseudovirus from infecting the cells. Ultimately, the expression of the pseudovirus within the cells can be quantified using a detection instrument to determine the neutralizing titer of the serum.

Table S1. Results for different cell generations.

Type of pseudovirus	Cell generations	*n*	GMT	GCV (%)
EV71	P3	3	3349	4.5
	P14	3	3355	7.9
	P31	3	5002	38.5
CA16	P3	3	2575	11.9
	P14	3	2080	12.0
	P31	3	2032	3.3
CA10	P3	3	2185	17.2
	P14	3	1931	6.8
	P31	3	2673*	16.0
CA6	P3	3	4056	25.6
	P14	3	3470	10.6
	P31	3	4479	5.2

n: number of independent experiments; GMT: geometric mean of NT_{50} values of pseudovirus-based neutralization assay; GCV: variability expressed using geometric coefficients of variation, GCV = $(10^{S}-1 \times 100\%$, where S is the standard deviation of the $\log_{10}$ transformed potencies. * indicates a statistically significant difference in the results between P14 and P31 (P=0.0411).

Table S2. Results for different neutralization incubation durations.

Type of pseudovirus	Neutralization incubation duration (h)	*n*	GMT	GCV (%)
EV71	1	3	5308	10.0
	2	3	6096	3.5
	3	3	6719	3.2
CA16	1	3	2651	6.8
	2	3	2976	5.7
	3	3	3599	11.5
CA10	1	3	4124	5.6
	2	3	3847	17.5
	3	3	3877	12.5
CA6	1	3	3386	5.9
	2	3	4186	6.6
	3	3	4821	10.2

n: number of independent experiments; GMT: geometric mean of NT_{50} values of pseudovirus-based neutralization assay; GCV: variability expressed using geometric coefficients of variation, GCV = $(10^S-1) \times 100\%$, where S is the standard deviation of the $\log_{10}$ transformed potencies.

Table S3. Results for intermediate precision.

Serum	NtAb titer (U/mL)	Experimenter	*n*	GMT	GCV (%)
Anti-EV71	2700	A	3	7248	20.9
		B	3	7549	
	1350	A	3	3249	11.9
		B	3	3177	
	675	A	3	1859	12.8
		B	3	1661	
	338	A	3	693	15.6
		B	3	794	
	169	A	3	375	20.7
		B	3	487	
Anti-CA16	2700	A	3	10768	7.9
		B	3	10709	
	1350	A	3	5794	10.9
		B	3	5698	
	675	A	3	2662	7.2
		B	3	2498	
	338	A	3	1351	12.5
		B	3	1407	
	169	A	3	743	31.1
		B	3	463	
Anti-CA10	3700	A	3	4026	5.3
		B	3	3727	
	1850	A	3	1916	4.9
		B	3	1854	
	925	A	3	1004	6.7
		B	3	916	
	463	A	3	442	4.8
		B	3	457	
	231	A	3	246	4.9
		B	3	229	
Anti-CA6	1500	A	3	12511	16.2
		B	3	13364	
	750	A	3	6074	12.3
		B	3	5534	
	375	A	3	2968	9.8
		B	3	2752	
	188	A	3	1410	9.0
		B	3	1247	
	94	A	3	677	11.1
		B	3	666	

NtAb titer: titer of neutralizing antibodies (NtAbs) in each serum sample estimated using the cell-based neutralization assay with the national standards for NtAbs against EV71 (NIFDC code: 300017), CA16 (NIFDC code: 300030), CA10 (NIFDC code: 300044), and CA6 (NIFDC code: 300043); *n*: number of independent experiments; GMT: geometric mean of NT_{50} values of

pseudovirus-based neutralization assay; GCV: variability expressed using geometric coefficients of variation, GCV = (10S−1) × 100%, where S is the standard deviation of the $\log_{10}$ transformed potencies.

Table S4. Results for accuracy.

Serum	NtAb titer (U/mL)	*n*	PBNA NT_{50}		Relative bias (%)
			Theoretical value*	Measured value	
EV71	1350	6	3699	3213	−13.2
	675	6	1850	1760	−4.8
	338	6	925	744	−19.6
	169	6	462	431	−6.8
CA16	1350	6	5369	5746	7.0
	675	6	2685	2580	−3.9
	338	6	1342	1379	2.7
	169	6	671	603	−10.1
CA10	1850	6	1938	1885	−2.8
	925	6	969	960	−0.9
	463	6	485	449	−7.3
	231	6	242	238	−2.0
CA6	750	6	6469	5804	−10.3
	375	6	3234	2860	−11.6
	188	6	1617	1328	−17.9
	94	6	809	672	−16.9

NtAb titer: titer of neutralizing antibodies (NtAbs) in each serum sample estimated using the cell-based neutralization assay with the national standards for NtAbs against EV71 (NIFDC code: 300017), CA16 (NIFDC code: 300030), CA10 (NIFDC code: 300044), and CA6 (NIFDC code: 300043); *n*: number of independent experiments; GMT: geometric mean of NT_{50} values of pseudovirus-based neutralization assay; Relative bias: (measured value − theoretical value)/theoretical value × 100%. * The theoretical value is the expected NT_{50} result following the appropriate dilution of a known NT_{50} antiserum.
